# Correlates of meal skipping in young adults: a systematic review

**DOI:** 10.1186/s12966-016-0451-1

**Published:** 2016-12-01

**Authors:** Felicity J. Pendergast, Katherine M. Livingstone, Anthony Worsley, Sarah A. McNaughton

**Affiliations:** Institute for Physical Activity and Nutrition (IPAN), School of Exercise and Nutrition Sciences, Deakin University, 221 Burwood Highway, Burwood, Geelong, Victoria 3125 Australia

**Keywords:** Meal skipping, Young adults, Correlates, Systematic review, Eating behaviour

## Abstract

**Background:**

Meal skipping rates may be highest during young adulthood, a period of transition and development. Although these dietary behaviours may increase future risk of chronic disease, limited research has investigated correlates of meal skipping in young adults.

**Methods:**

A systematic literature search was conducted to identify studies that investigated correlates of meal skipping behaviours in young adults (aged 18–30 years). EBSCO host, MEDLINE Complete, Global Health, Scopus, EMBASE, Web of Science and Informit platforms were searched for eligible articles. Correlates were defined as any factor that was either associated with meal skipping or was self-reported by the participant to have an influence on meal skipping. Randomised controlled trials, prospective cohort studies, case-control studies, nested case-control studies, cross-sectional studies, and longitudinal studies were eligible for inclusion.

**Results:**

Three-hundred and thirty-one articles were identified, 141 full-text articles assessed for eligibility, resulting in 35 included studies. Multiple methodological and reporting weaknesses were apparent in the reviewed studies with 28 of the 35 studies scoring a negative rating in the risk of bias assessment. Meal skipping (any meal), defined as the skipping of any meal throughout the day, was reported in 12 studies with prevalence ranging between 5 and 83%. The remaining 25 studies identified specific meals and their skipping rates, with breakfast the most frequently skipped meal 14–88% compared to lunch 8–57% and dinner 4–57%. Lack of time was consistently reported as an important correlate of meal skipping, compared with correlates such as cost and weight control, while sex was the most commonly reported associated correlate. Breakfast skipping was more common among men while lunch or dinner skipping being more common among women.

**Conclusions:**

This review is the first to examine potential correlates of meal skipping in young adults. Future research would benefit from stronger design and reporting strategies, using a standardised approach for measuring and defining meal skipping.

**Electronic supplementary material:**

The online version of this article (doi:10.1186/s12966-016-0451-1) contains supplementary material, which is available to authorized users.

## Background

Young adulthood, is a unique developmental phase defined as a period of multiple transitions and the development of independence [1, 2]. During this time, individuals develop the skills needed to engage and practice behaviours, such as healthy eating, that track into later life [3, 4]. Research suggests that young adults engage in poor eating behaviours, such as low fruit and vegetable consumption [4, 5], high consumption of energy-dense snack foods [6], and frequently fail to consume regular meals [7, 8].

Meal skipping is the omission or lack of consumption of one or more of the traditional main meals (breakfast, lunch or dinner) throughout the day [9]. The regular omission of meals, particularly the breakfast meal, has been associated with poorer diet quality [10], lower intakes of total energy, vitamins and minerals [11–13], increased risk of central adiposity [14, 15], markers of insulin resistance [15, 16] and cardio metabolic risk factors [15, 17]. Estimated prevalence rates of meal skipping in the young adult population vary between 24 and 87% [18, 19], with young adults consistently reporting higher rates of meal skipping compared with other age groups [5]. Recent data from the Australian Health Survey 2011/12 showed that 39% of Australian young adults (19–24 years) reported eating breakfast less than 5 days per week, compared with 10% children (8–11 years) and 33% of all adults (>18 years) [5]. Despite the significant health implications of meal skipping and its higher prevalence among young adults, limited research has investigated correlates of this unhealthy eating behaviour.

Conceptual models or frameworks are useful in understanding and explaining correlates of eating behaviours such as meal skipping. While there are a number of possible conceptual frameworks in the literature, this review will use the framework developed by Story et al. [20] to categorise the correlates of meal skipping. This framework combines ecological perspectives with social cognitive theories (SCT) resulting in a framework which takes into account the relationship between people and their environments seen in ecological models [21], and socio-environmental, personal and behavioural factors seen within SCT [22]. This social-ecological framework (SEF) is made up of the following four domains: 1) Individual influences (intrapersonal); 2) Social environmental influences (interpersonal); 3) Physical environmental influences (community settings); 4) Macrosystem influences (societal) [20], and has been used effectively in eating behaviour research [20, 23]. Previous research investigating correlates of meal skipping in various populations have identified correlates from each of these domains. Individual influences such as smoking status [24] and infrequent physical activity [25]; social environmental influences of family support [24]; and physical environmental influences such as housing type have been associated with meal skipping behaviours [26].

Given the importance of this life stage in the development of long term health behaviours and the high prevalence of poor eating behaviours in this population group, understanding the correlates of meal skipping is needed to better inform public health strategies and dietary interventions. However, there have been no systematic reviews to synthesise the evidence on correlates of meal skipping. Therefore, the aim of the present review was to systematically evaluate the literature on correlates of meal skipping in young adults (18–30 years) using a SEF.

## Methods

### Protocol

This study followed the procedures for systematic review reporting as described by the Preferred Reporting Items for Systematic Reviews and Meta-Analysis (PRISMA) (Additional file 1) recommendations [27].

### Search strategy

A systematic and comprehensive search of the literature surrounding meal skipping was conducted in January 2016. The search was limited to human studies, published in English post 1979. This time frame was chosen, as the first studies describing eating patterns were published in early 1980’s [28, 29]. Academic Search complete, CINAHL Complete, PsycINFO, SocINDEX, ERIC and Education Source were searched through EBSCO Host. MEDLINE Complete, Global Health, Scopus, EMBASE, Web of Science and Informit searches were conducted independently. Bibliographies of included articles were also reviewed (hand searched) for additional articles. Search terms were tested prior to the recorded search to ensure that appropriate articles were identified. The following search terms were used during the systematic searching of databases: (Meal skipping, meal frequency, meal omission (skip* OR frequen* OR omission*) N5 meal*) AND (Young adults, emerging adult, college students (young OR emerge*) N5 adult* (college OR university* OR undergraduate* OR “post-secondary*” OR postgraduate*) N5 student*)) AND (Eating habits, feeding habits, food habits, diet habits, meal habits (diet* OR eat* OR meal* OR feed* OR food*) N5 habit*).

### Eligibility criteria

To be included in this review each article was required to meet the following criteria: (1) original research article, published in a peer-reviewed journal, with full text in English language; (2) the study participants were young adults aged 18–30 years or with a mean age between 18 and 30 years, or aged 18–30 years at baseline for longitudinal studies, for studies that did not report a mean age, the participants needed to be referred to as university or college students; (3) the study participants were free from disease and were community-dwelling; (4) there was a measure of meal skipping, meal omission, or meal frequency reported (assessed as any meal skipped throughout the day or according to meal type e.g. breakfast, lunch, dinner, and supper); (5) there was at least one meal skipping correlate reported; (6) the study design was one of the following: randomised controlled trial, prospective cohort study, case-control study, nested case-control study, cross-sectional study, longitudinal study.

Articles were excluded if they met any of the following criteria: (1) studies published as abstracts, conference proceedings, posters or not in the English language; (2) the article included specific populations of young adults (e.g. athletes, institutionalised populations); (3) the participants’ mean age was outside the range of 18–30 years or included children or older adults; (4) there was no measure or report of meal skipping, meal omission, or meal frequency; (5) there was no correlate of meal skipping reported; (6) the study design was one of the following; case reports, opinion articles, reviews, narrative reviews, systematic reviews, meta-analyses.

### Study selection

Two reviewers (FJP and KML) independently assessed titles and abstracts for eligibility. Any articles that did not meet eligibility criteria were excluded. Full texts of the remaining articles were then obtained and screened for inclusion. If consensus between reviewers was not reached a third reviewer (SAM) was consulted and a consensus approach was used.

### Data extraction and synthesis

Data extraction was initially conducted by one reviewer (FJP) using an electronic spreadsheet. Information extracted included author, year, country, design, sample characteristics, participants’ age, how meal skipping was measured, definition of meal skipping; frequency of meal skipping and correlates of meal skipping. Following the initial data extraction the spreadsheet was verified for accuracy and consistency by a second reviewer (KML) with consensus reached with the help of the third reviewer (SAM) in cases of disagreement.

### Quality and risk of bias assessment

All included studies were assessed for quality and risk of bias by two independent reviewers (FJP, KML) using the Academy of Nutrition and Dietetics Quality Criteria Checklist [30]. Any discrepancies between the reviewers was resolved by discussion and consensus with a third reviewer (SAM). Articles were assessed against 10 criteria and were assigned a positive, negative or neutral rating. As per guidelines, an article was deemed positive if criteria 2, 3, 6, 7 and at least one additional criterion was awarded a ‘yes’, neutral if criteria 2, 3, 6 and 7 did not score a ‘yes’, or negative if more than six of the criteria were awarded a ‘no’. Each criteria was assessed as either ‘yes’ or ‘no’. Criteria included: (1) the study clearly stated the research question; (2) the selection of participants was free from bias; (3) if study groups were comparable; (4) participant withdrawal process was documented; (5) the use of blinding was documented; (6) participant compliance was measured; (7) the measurements used were valid and reliable; (8) appropriate statistical analysis was used; (9) biases and limitations were documented (10) funding or sponsorship was reported.

## Results

**Fig. 1:**
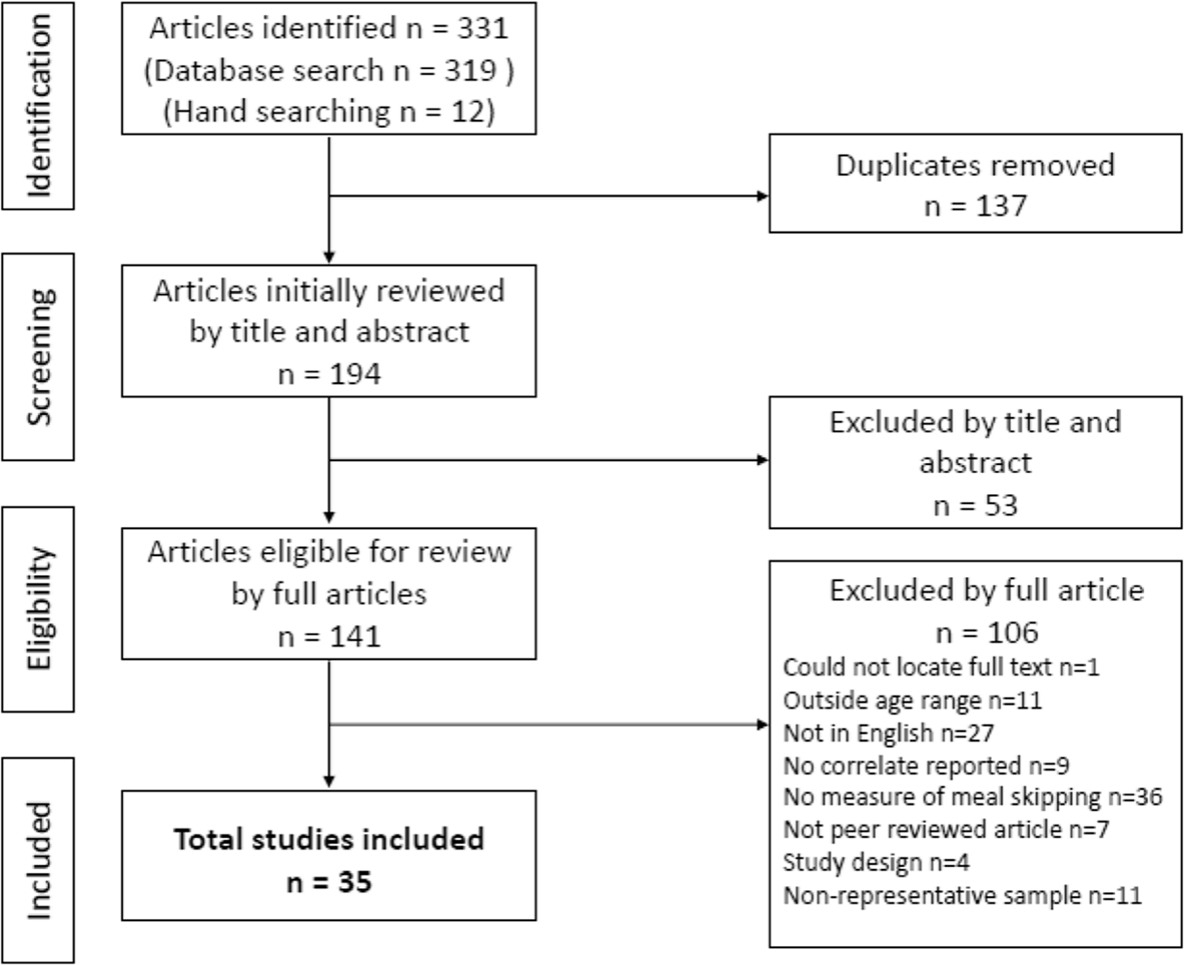
Flow chart summary of articles identified in search and included in review

The study selection process, including reasons for excluding studies is summarised in Fig. 1. Of the 331 articles identified, 194 articles were screened based on their title and abstract. Of these, 141 full-text articles were assessed for eligibility and 35 studies were included in the review. Study characteristics and risk of bias scores are presented in Table 1 and Additional file 2 respectively. Risk of bias assessment indicated four studies had a positive rating (low risk of bias) [1, 31–33], 28 studies had a negative rating (high risk of bias) [12, 34–60] and three studies had a neutral rating [61–63].

**Table 1:** Characteristics of the included studies

Reference, year	Country	Design	Sample characteristics, N (% women)	Participants; age, years (mean ± SD)	How was meal skipping measured?	Definition of meal skipping	Frequency of meal skipping	Correlates of meal skipping
1. Afolabi et al. 2013 [34]	Nigeria	Cross-sectional	University students; 140 (40% F)	N/R	Q: Do you skip meals? *(Yes/No)* Q: Why do you skip meals?	“Yes”; Text	53.6% M skipped meals, 35% F skipped meals	Reasons for skipping meals: -Time 48%, -Appetite 19%, -Cannot cook 13%, -Illness 3%, -Money 6%
2. Akarslan et al. 2008 [61]	Turkey	Cross-sectional	Young adults; 416 (59% F)	18-25 years; (23.2 ± 0.97 years)	Q: Frequency of B, L, D? *(Almost always/Sometimes/Very seldom/Never in a one year period)*	N/R	70% had regular main meals. Regular B 69.2%, Regular L 75.5%, Regular D 76.0%	SEX: (0 TMS)
3. Aryee et al. 2013 [35]	Ghana	Cross-sectional	Nurses; 220; (66% F)	20–60 years; (67.3% 20–30 years)	Q: Do you meal skip? *(Yes/No)*	“Yes”	53.6% skipped meals	BMI: (+TMS)
4. Bahl et al. 2013 [36]	USA	Cross-sectional	College students (Business); 353 (43% F)	N/R	Q: How many days during the last week (0–7) did you skip meals?	Numerical	Participants who had mindfulness training skipped meals on 1.25 days during the last week compared to 1.94 days for the non-training group	MINDFULLNESS: (- TMS) SATISFIED: (quality of life - TMS)
5. Beerman et al. 1990 [62]	USA	Cross-sectional	College students (Nutrition); 152 (56% F)	74% ≤ 21 years	Q: Do you skip meals? *(Regularly/Rarely)*	“Regularly”	66% of on or off campus students skipped meals. 36% of those living in Greek housing skipped meals.	LIVING SITUATION: (Greek housing -TMS) SEX: (0 TMS) Reasons for meal skipping: -Time 61%
6. Chung et al. 2003 [37]	Korea	Cross-sectional	College students; 180 (100% F)	20.41 ± 1.82 years	Q: What is your breakfast status? *(Rarely eating, Frequently eating or Daily eating)*	“Rarely eating” or “Frequently eating”	74.4% skipped B	BMI: (0 TMS)
7. Coli*ć* Bari*ć* et al. 2003 [38]	Croatia	Cross-sectional	University students; 2075 (53% F)	21.7 ± 2.0 years	Specially designed FFQ	Numerical; Regular B, defined as having B 6 or 7 times per week	B consumed on 3.4 days/week, L 6 days/week, D 4.7 days/week. 32.2% F and 25.7% M consumed B regularly	SEX: (F + L, +D) BMI: (+BS) EXERCISE: (3.5 h versus 2.6 h of exercise per week -BS)
8. Danquah et al. 2010 [39]	Ghana	Cross-sectional	University students; 150 (75% F)	64.6% 21–30 years	Q: Do you eat breakfast? *(Yes/No)*	“No”	25% skipped B, 8% skipped L, 5% skipped D	ETHNIC: (Caucasian -BS) SEX: (F + BS) AGE: (15–20 years -BS) COURSE TYPE: (Science students + BS) Reasons for skipping B: -No time (57%), -Not hungry (22%), -Eat late at night (5%), -Busy schedule (3%), -No reason (13%). Reasons for skipping L: -No time (50%), -Not hungry (25%), -No reason (3%), Reasons for skipping D: -No time (38%), -Busy schedule (12%), -Watching my weight (50%)
9. Deepika 2015	India	Cross-sectional	College students; 120 (80% F)	18–23 years	Q: Do you A) Take all three meals, B) Skip meals with substitute or C) Skip meals without substitute?	“B” or “C”	83.3% skipped meals	Reason for skipping meals: -Time 40%, -Taste 30%, -Social desirable 28.3%, - Habit 1.7%
10. Eittah 2014 [41]	Egypt	Cross-sectional	University students (Nursing); 300 (100% F)	17–22 years; (20.05 ± 1.62 years)	Q: Do you always neglect to eat - B, L, D? *(Yes/No)*	“Yes”	72.7% skipped B, 7.3% skipped D, 6% skipped B and D	MENSTRUAL REGULARITY: (Menstrual regularity -BS)
11. Eldisoky, 2003 [42]	Saudi Arabia	Cross-sectional	University students; 61 (100% F)	19–24 years	Q: Do you usually have B? *(Yes/Sometimes/No)*	“Sometimes” or “No”	63% skipped B, 61% skipped L, 31% skipped D	MOTHERS EDUCATION LEVEL: (0 TMS) Reasons for skipping meals: -Hunger 48%, -Time 31%, -Weight control 21%
12. Evagelou et al. 2014 [43]	Greece	Cross-sectional	University students (Nursing); 435 (83.4% F)	N/R	Q: N/R *(Rarely/At least one/Two/Three/Four/Five/Six/Seven times a week)*	N/R	31% skipped B	SEX: (0 BS)
13. Freedman 2010 [63]	USA	Cross-sectional	College freshman; 756 (61% F)	N/R	Q: Frequency of meal intake? *(Never/1-3 times a week/4-6 times a week/7 days a week)*	“Never”	24.7% skipped B, 7% skipped D	LIVING SITUATION: (On campus + BS) SEX: (F + DS) ETHINICITY: (Caucasian -BS, -DS)
14. Huang et al., 1994 [44]	USA	Cross-sectional	College students (Nutrition); 1912 (68% F)	M 20 years, F 19 years	1 day-food record (weekday)	Meal not reported in food record	22% skipped B, 8% skipped L, 5% skipped D	SEX: (0 TMS)
15. Kapinos & Yakusheva, 2011 [31]	USA	Longitudinal	University students; 388 (63% F)	18.1 years	Q: Over the past year, how many meals per day did you typically eat?	Numerical	2.88 meals/day at baseline, 2.61 meals/day one year later	ENROLLING IN UNI: (Second year of uni + TMS) LIVING SITUATION: (M living in dormitories with a dining hall -TMS)
16. Kim et al., 2010 [45]	China	Cross-sectional	First year University students; 2427 (63.4% F)	18.9 years	Q: Have you in the past month skipped meals? *(Often/Occasionally/Never)*	N/R	Skipped meals at least monthly in past year -16.2%, Skipped meals in the past week - 4.8%	INTERNET USE: (4 + hours/day + TMS)
17. Lamia Dhia & Ban Faud 2014 [46]	Iraq	Cross-sectional	University students; 350 (Sex NR)	N/R	Q: No of meal/day? Q: If missed what is the most missed meal?	Numerical; Text	51.1% consumed < 3 meals/day. Of those who missed a meal 88.5% skipped B, 11.5% skipped D	Reasons for meal skipping: -Time
18. Laska et al. 2010 [1]	USA	Cross-sectional	Young adults; 1687 (56% F)	18–23 years; (20.5 years)	Q: How often do you eat B, L, D during the past week? *(Never/1–2 d/3–4 d/5–6 d/Every day)*	Numerical	B consumption ranged from 2.7 to 3.5 days/week, L 5.3–5.8, D 6.1–6.5.	LIVING SITUATION: (Living with parents + BS, +DS)
19. Lee & Yoon 2014 [47]	Korea	Cross-sectional	University students (Food and Nutrition 50.3%); 159 (62.3% F)	18–28 years; 56% 18–20 years	Q: Missed meal? Q: Reason of skipping meal?	Text; Text	83.6% skipped B, 6.9% skipped L, 8.2% skipped D	AGE: (18–20 years + TMS) Reason for skipping B: -Time 61%, -Habit 17.6%, -Appetite 11.9%
20. Musaiger & Radwan 1995 [48]	United Arab Emirates	Cross-sectional	University students; 215 (100% F)	18–30 years; (19.7 ± 1.3 years)	Specially designed questionnaire on meal pattern	N/R	15.8% skipped B, 11.2% skipped L, 7% skipped D	BMI: (0 TMS)
21. Neslisah & Emine 2011 [49]	Turkey	Cross-sectional	University students; 400 (42% F)	19–24 years; (21.7 ± 1.8 years)	1 × 24 h diet record	Meal not reported in food record	47.7% skipped B, 25.2% skipped L	SEX: (M+ BS) (F + LS)
22. Nicklas et al. 1998 [12]	USA	Cross-sectional	Young adults; 504 (58% F)	19–28 years; (23 years)	1 × 24 h diet recall	B had to equal or exceed macronutrient value of 1 serving of milk.	37% skipped B	ETHNICITY: (0 BS) SEX: (0 BS)
23. Nzeagwu & Akagu 2011 [50]	Nigeria	Cross-sectional	University students; 342 (63% F)	16–25 years; 81% 20–25 years	Q: What meal do you usually skip? Q: Why do you skip meals?	Text; Text	27.8% skipped B, 16.9% skipped L, 5.6% skipped D, 4.4% skipped B and L, 2.3% skipped B and D, .5% skipped L and D	Reasons for skipping meals: -Time 40.5%, -Fasting/religion 6.7%, -Weight control 10.4%, -Money 9.9%
24. Ozilgen 2011 [51]	Turkey	Cross-sectional	University students; 408 (56% F)	18–24 years	Q: How many times a day do you eat B, L, and D?	Numerical	~80% F skipped meals, ~72% M skipped meals, ~77% M skipped B, ~61% F skipped B	SEX: (M + BS)
25. Sakamaki et al. 2005 [52]	Japan and Korea	Cross-sectional	University students (141 Korean), 124 Japan); 265 (100% F)	(20 ± 1.8 years)	Q: Do you always take breakfast? *(Daily/3-4 times per week/Once or twice per week/Rarely)*	Everything except “Daily”	21% Japanese skipped B, 64% Korean skipped B	ETHNICITY: (Japanese –BS)
26. Sato-Mito et al. 2011 [32]	Japan	Cross-sectional	University students living at home (Dietetics); 3304 (100% F)	18–20 years; (18.1 ± 0.3 years)	Q: During the previous month how many meals have you skipped?	Numerical	B skipped 1.00 ± 1.74 times/week, L skipped 0.20 ± 0.73 times/week, D skipped 0.32 ± 1.09 times/week	SLEEP: (Feel asleep later in the night + TMS)
27. Shimbo et al. 2004 [53]	Japan	Cross-sectional	University Students; 71 (100% F)	19–23 years;	1 × 24 h food duplicate portion samples	Meal not reported in food record	14% skipped B	LIVING SITUATION: (Living away from home + BS)
28. Suliburska et al. 2012 [54]	Poland	Cross-sectional	Young adults; 600 (50% F)	18 years	Q: How many meals do you consumed in a typical day of the week?	Numerical	5% of overweight ate >2 meals/day, 9% of normal weight ate >2 meals/day	BMI: (Overweight –TMS) SEX: (F-TMS) LIVING SITUATION: (Rural + TMS)
29. Suliga et al. 2012 [55]	Poland	Cross-sectional	University students; 925 (100% F)	N/R	Q: Frequency of main meals (B, L, D, and S)? *(Daily/2-6 times a week/Rarely)*	“Rarely”	11% rarely ate B, 8.2% rarely ate L, 12.6% rarely ate D	BODY WEIGHT SELF PERCEPTION: (+ TMS)
30. Tanaka et al. 2008 [33]	Japan	Cross-sectional	University students (Medicine); 127 (30.4% F)	(20.5 ± 0.8 years)	Q: B consumption *(Every day/Not every day/Completely skipping everyday)*	“Completely skipping everyday”	15.7% skipped B	FATIGUE: (+BS)
31. Tominaga et al. 2012 [56]	Japan, Korea and Austria	Cross-cultural	University students; 276 Japan, 103 Korea, 127 Austria, (100% F)	Japan; (19.9 ± 1.2 years), Korea (21.5 ± 1.8 years), Austria (22.3 ± 5.2 years)	Q: Frequency of B, L, D *(Never/Occasionally/Sometimes/Almost every day)*	“Never” or “Occasionally” or “Sometimes”	JAPAN - 50% skipped B, 15% skipped L, 17% skipped D. KOREA - 54% skipped B, 51% skipped L, 46% skipped D. AUSTRIA - 42% skipped B, 29% skipped L, 47% skipped D	ETHNICITY: (Austrian –BS), (Japanese –LD and DS)
32. Ukegbu et al. 2015 [57]	Nigeria	Cross-sectional	University students; 200 (47% F)	16–25 years	2 × 24 h recalls. Consecutive days including a weekend day.	Meal not reported in food record	41.5% skipped B, 21.5% skipped L, 7% skipped D	Reason for meal skipping: -Time 42.5%, -Weight control 23.5%, -Fasting/religion 21.5%, -Money 12.5%
33. Yahia et al. 2008 [58]	Lebanon	Cross-sectional	University students; 220 (56.4% F)	(20 ± 1.9 years)	Q: Do you take breakfast? *(Daily/3-4 times per week/1-2 per week/Rarely)*	“Rarely”	67% skipped B	SEX: (M + BS)
34. Yildiza et al. 2011 [59]	Turkey	Cross-sectional	University students (Medical); 301 (100% F)	18–25 years; (21.2 ± 1.7 years)	Q: Frequency of B, L, D? *(Never/Occasionally/Most of the days/Everyday)*	“Never” or “Occasionally” or “Most of the days”	74% skipped B, 57% skipped L, 37% skipped D, 69.7% were skipping at least one meal per day.	Reason for meal skipping: -Time 46.7%
35. Yilmaz et al. 2014 [60]	Turkey	Cross-sectional	University students (Medical); 995 (48% F)	M (21.25 ± 1.97 years); F (20.94 ± 1.77)	Q: I usually skip meals? *(Yes/No)*	“Yes”	35.8% skip B, 27.9% usually skip meals	DEPRESSION: (+BS)

*N/R* not reported, *M* males, *F* females, *B* breakfast, *L* lunch, *D* dinner, *BMI* body mass index, *FFQ* food frequency questionnaire, *BS* Breakfast skipping, *LS* Lunch skipping, *DS* Dinner skipping, *TMS* Total meal skipping, (0) = No association, (+) = Positive association, (-) = Negative association

### Study characteristics

The included studies were conducted in 15 countries: seven from the United States [1, 12, 31, 36, 44, 62, 63], five from Japan [32, 33, 52, 53, 56] five from Turkey [49, 51, 59–61] three from Nigeria [34, 50, 57] two from Korea [37, 47], Ghana [35, 39] and Poland [54, 55] and one from China [45], Croatia [38], Egypt [41], Greece [43], India [40], Iraq [46], Saudi Arabia [42] and the United Arab Emirates [48].

Of the 35 included studies, 17 consisted of primarily female participants (>50%) [1, 12, 31, 35, 38–40, 43–45, 47, 50, 51, 58, 61–63], 10 included female participants only [32, 37, 41, 42, 48, 52, 53, 55, 56, 59], six consisted of primarily male participants (>50%) [33, 34, 36, 49, 57, 60]. One study had an even distribution of male and female participants [54], and one study failed to report the sex distribution of its participants [46].

All but one of the included studies were cross-sectional; the longitudinal study collected data at two times points approximately 18 months apart [31]. The included studies used a variety of different methods to assess and define meal skipping. Dietary intake methods were used by six studies; four used 24 h diet recalls [12, 49, 53, 57], one used food records [44], and one used a specially designed food frequency questionnaire (FFQ) [38]. This specially designed FFQ collected information on the self-reported number of meals and snacks consumed daily. Meal skipping was defined as a non-reported meal within the respective dietary assessment method. Binary response questions were also used to measure meal skipping, with options *“Yes/No”* or *“Regularly/Rarely”* used by eight studies [34, 35, 39, 41, 47, 50, 60, 62]. Question wording influenced how meal skipping was defined in these binary response questions. Meal skipping was measured numerically by seven studies [1, 31, 32, 36, 46, 51, 54], and categorically by 14 studies [33, 37, 40, 42, 43, 45, 48, 52, 55, 56, 58, 59, 61, 63]. These studies had varying response options and used varying cut points to define meal skipping. Five of the 14 studies did not report how they defined meal skipping from their response categories [43, 45, 48, 55, 61].

Meal skipping (any meal) was reported in 10 studies [31, 34–36, 40, 45, 51, 54, 55, 62] and ranged from 4.8 to 83.3%, while the remaining 25 studies identified specific meals and their skipping rates. All 25 of these studies reported breakfast skipping with prevalence rates of ranging from 14 to 88.5%. Lunch skipping was reported by 11 studies [39, 42, 44, 47–50, 56, 57, 59, 61] with rates ranging from 8 to 57%. Dinner or supper skipping was reported in 13 studies, [39, 41, 42, 44, 46–48, 50, 56, 57, 59, 61, 63] with rates ranging from 5 to 47%.

The majority of studies (28 of the 35 studies) examined correlates by examining associations between factors and meal skipping behaviours through Chi-square, One-way ANOVA, Duncan’s’ multiple range test and regression (linear and logistic) statistical analysis [1, 12, 31–33, 36–39, 41–45, 47–49, 51–56, 58, 60–63]. Another approach used to examine correlates of meal skipping (used in 10 studies), was the use of a ranking methodology where participants were asked to rank potential correlates against other meal skipping correlates [34, 39, 40, 42, 46, 47, 50, 57, 59, 62]. From these ten studies, ten ranked correlates were reported.

### Individual influences (Intrapersonal)

Of the 35 studies included in this review, 33 studies assessed correlates from the SEF that could be considered intrapersonal correlates. These included sex, age, ethnicity, body mass index (BMI), education, menstrual regularity, physical activity, internet use, and a list of cognitive influences.

#### Sex

Sex was reported as a correlate of meal skipping by 12 studies; three reported meal skipping (any meal) [44, 54, 61] and nine reported on specific meal skipping [12, 38, 39, 43, 44, 49, 51, 58, 63]. Two studies identified no difference in meal skipping (any meal) in relation to sex [44, 61], while one study reported meal skipping (any meal) to be more likely in males [54]. Two studies reported no significant difference in breakfast skipping between sexes [12, 43], three reported breakfast skipping to be more likely in males [49, 51, 58], while two reported breakfast skipping to be more likely in females [39, 44]. However, Huang et al. [44] reported that females were more likely to skip breakfast in summer months, this associations was not present in winter months. Two studies reported lunch skipping [38, 49], and two dinner skipping [38, 63], both studies found females to be more likely to skip these meals (lunch [38, 49] and dinner [38, 63]) compared to males.

#### Age

Two studies reported an association between age and breakfast skipping [39, 47]. Danquah et al. [39], reported breakfast skipping to be more likely in those aged 15–20 years when compared to those aged 21–30 years. While, Lee and Yoon [47] reported meal skipping (any meal) to be more likely in those aged 18–20 years compared to those aged 24–28 years.

#### Ethnicity

Ethnicity was reported to be associated with breakfast skipping in five studies [12, 39, 52, 56, 63]. Of the studies that included Caucasian participants [12, 39, 56, 63], three found breakfast skipping to be more likely in those who were Caucasian compared with other ethnicities (Japanese, Korean, African American) [39, 56, 63], and one found no association [12]. Another study found breakfast skipping and meal skipping (any meal) to be more likely in Korean young adults compared with Japanese young adults [52]. While, lunch and dinner consumption was found to be more common in Japanese young adults compared to Caucasian and Korean young adults [56].

#### Body mass index (BMI)

Five studies reported that BMI was associated with meal skipping [35, 37, 38, 48, 54]. Meal skipping (any meal) was reported in four studies, two found no association between BMI and meal skipping (any meal) [37, 48], one reported meal skipping (any meal) to be more likely in those with an increased BMI [35], while Suliburska [54] found meal skipping (any meal) less likely in those with an increased BMI. Breakfast skipping was reported by one study and was more likely with those with an increased BMI [38].

#### Education

Three studies examined education and its association with meal skipping behaviours. Eldisoky [42], reported maternal education status and its relationship with breakfast skipping, although this was not significant. Kapinos & Yakusheva reported those in second year university were more likely to report meal skipping (any meal) compared to those in first year university [31]. While, Danquah et al. [39], reported those in science courses were more likely to report breakfast skipping compared to students enrolled in humanities courses.

#### Menstrual regularity

Eittah [41] found breakfast skipping to be more likely in those with an irregular menstrual cycle compared to those with a regular menstrual cycle.

#### Physical activity

Colić Barić et al.[38], found breakfast consumption (6 or 7 times per week) was more likely in those who spent ≥ 3.5 h exercising per week when compared to those who did 2.6 h per week.

#### Internet use

One article reported meal skipping (any meal) to be more common in those who used the internet heavily (>4 h/day) [45].

#### Cognitive influences

##### Fatigue

Two studies examined the association between fatigue and meal skipping [32, 33]. Tanaka et al. [33] found breakfast skipping to be more likely in those experiencing fatigue, while Sato-Mito et al. [32] found meal skipping (any meal) to be more likely in those who’s mid-point in sleep was later (falling asleep after 1.30 AM and the mid-point of sleep falling at 5.31 ± 0.55 AM).

##### Psychological wellbeing

Three studies documented associations between psychological factors and meal skipping (any meal). Yilmaz et al. [60] found meal skipping (any meal) to be more likely in those with depressive symptoms; Suliga et al. [55], found meal skipping (any meal) to be more likely in those with a self-perception of being overweight; Bahl et al. [36] found meal skipping (any meal) to be less likely in those who were mindful, and meal skipping (any meal) to be less likely in those who had increased body satisfaction.

##### Time

Time or the lack of time was mentioned in 10 studies and when considered against other correlates, time was ranked as the strongest perceived correlate of meal skipping in nine of the 10 studies [34, 40, 42, 46, 47, 50, 57, 59, 62].

##### Hunger

A lack of hunger was reported in four studies and ranged in importance from being the strongest correlate of meal skipping to the 3^rd^ strongest perceived correlate [34, 39, 42, 47].

##### Weight control

Weight control was discussed in four studies and ranged from being the strongest perceived correlate of dinner consumption to the 3^rd^ strongest perceived correlate of meal skipping (any meal) [39, 42, 50, 57].

##### Money

Money or the lack of money, was reported in three studies and was ranked as either 3^rd^ or 4^th^ strongest perceived correlate of meal skipping (any meal) [34, 50, 57].

##### Habit

Dietary habit was reported in two studies, one study ranked habit as the 2^nd^ strongest perceived correlate of breakfast skipping and the other ranked it as the 4^th^ strongest perceived correlate of meal skipping (any meal) [40, 47].

##### Religion

Fasting/religion was reported in two studies, where it was ranked as being either the 3^rd^ or 4^th^ strongest perceived correlate of meal skipping (any meal) [50, 57].

##### Taste

Taste was reported as being a correlate of meal skipping (any meal), with one study ranking it as its 2^nd^ strongest perceived correlate [40].

##### Cooking skills

Lack of cooking skills was reported by one study as 3^rd^ strongest perceived correlate of meal skipping (any meal) [34].

### Social environmental influences (Interpersonal)

Of the 35 studies included in this review, only one study assessed a correlate that could be considered to be part of the social environmental domain. The variable examined was the notion of “being sociable”. It appears to assess participants’ preference for prioritising social activities over eating, and participants ranked it as the 3^rd^ strongest perceived correlate of meal skipping (any meal) [40].

### Physical environment influences

Of the 35 studies included in this review, six studies assessed correlates that could be considered physical environmental correlates. These included rural/urban living environments and housing type.

#### Rural/urban living environment

Meal skipping (any meal) was more likely in those who resided in a rural area compared to those who lived in an urban area [54].

#### Housing type

Five studies focused on specific living environments such as housing types. Kapinos & Yakusheva [31], reported meal skipping (any meal) to be more likely in those living in university/college dormitories. Similarly, Beerman et al. [62], reported meal skipping (any meal) to be more likely in those residing with parents or in university dormitories when compared to those living in Greek university housing (fraternity or sorority housing). Individual meal skipping events were reported in three articles [1, 53, 63]. Two articles reported breakfast skipping to be more likely in those who lived away from home [53, 63], while one found breakfast skipping to be more likely in those living by one’s self or with parents compared with living on campus [1]. This same article reported the same association for dinner skipping [1].

### Overview

In conclusion, majority of included studies (*n* = 33) examined correlates found within the intra-personal domain of the SEF, one study examined a perceived correlate from the interpersonal domain, with six studies examining correlates from the physical environment domain.

## Discussion

### Main findings

To our knowledge, this is the first systematic review to investigate correlates of meal skipping in young adults. This review identified that the prevalence of meal skipping among young adults ranged between 5 and 83%. The breakfast meal was the most frequently skipped meal in comparison to the lunch or dinner meal, with rates ranging from 14 to 88.5%. The perception of time or lack of time was consistently reported as an important correlate of this behaviour, with nine of ten studies rating time as the biggest correlate of meal skipping. Sex was the most commonly reported associated correlate of meal skipping: breakfast skipping was more common among men and lunch or dinner skipping being more common among women. However, the studies were difficult to compare because of inconsistencies in measurement tools and definitions of meal skipping.

### Breakfast skipping

This review identified that young adults skipped breakfast more frequently than other main meals. These results are consistent with studies of other age groups, with the breakfast meal frequently reported as the most commonly skipped main meal. A sample of American elderly participants reported the prevalence of breakfast skipping was highest (10.7%) when compared to lunch skipping (8.6%) and dinner skipping (5.8%) [64], with similar results seen in children and adolescent populations [65, 66]. Meal skipping was assessed in a sample of college students with the breakfast meal never/rarely consumed by nearly half of participants (44.2%), compared with lunch (3.5%) and dinner (2.3%) [67]. This highlights that different age groups experience meal skipping at different rates and that different meals are skipped at different proportions within each age bracket. It is however important to note that within the literature the breakfast meal is more frequently examined than either the lunch or dinner meals.

### Perception of time

The influence of time or the perceived lack of time was reported within all ten of the studies that assessed ranked correlates. Nine of the ten studies reported time as the biggest perceived influence on meal skipping when ranked against other important correlates of young adult meal skipping. The young adult age period is characterised by transition, including moving out of the family home, commencement of further education and/or starting a career [2]. These competing demands require young adults to learn a range of skills, including prioritising tasks and coping with these new environments [3]. These findings are confirmed by previous literature, with time scarcity being recognised as having a negative impact on a range of eating behaviours [68]. Deliens et al. [69] reported the impact of time scarcity on university students, with results suggesting students preferred to spend time on activities other than cooking, and highlighted the importance of short meal preparation times. Similar results are seen within adolescent populations, with 52% indicating that a lack of time in the morning was the main reason for skipping breakfast [70]. Therefore, perceived lack of time, may be a result of varied prioritisation with healthy eating behaviours poorly prioritised [69].

The notion of time is a temporal structure, self-reported by individuals and can have multiple interpretations and ramifications [71]. Psychologists’ such as Zimbardo have defined an individual’s time perspective as one of the most powerful influences on human behaviour [72]. However, none of the included articles provided a definition of time, which limited the ability of the present review to identify how time may have influenced meal skipping. For example, “time” may have been attributed to food shopping, preparation, cooking time or eating time and may have been interpreted differently by individuals. Given that the perception of time is underpinned by person-specific psychological constructs, the methodological approaches in the included studies were not detailed enough to provide a conclusive association with meal skipping. As the examination of time as a major barrier to health behaviours is complex, it requires stronger definitions and measurement tools before it is able to provide comparable and valid results. Further research is needed to examine, and develop an understanding of trade-offs and prioritisation of lifestyle factors seen between individuals.

### Sex and meal skipping

This review identified that meal skipping (any meal) and breakfast skipping were more likely in males, while lunch and dinner skipping were more likely in females. These associations are unlike those seen in other age groups, with adolescent studies reporting breakfast [7, 25, 66, 70] and lunch [66] skipping more likely in females. The results seen within this review however, are consistent with other research in this age group, with results in undergraduates student samples (not eligible for this review) finding no association between sex and breakfast skipping [73], and females more likely to skip dinner [74].

Results across included studies varied, with many studies samples dominated by a single sex. Direct comparisons between sexes become limited when sample sizes are heavily skewed towards one particular sub group. Previous literature looking at the difference between sexes and eating behaviours reports significant differences in food choices between sexes; females generally have higher intakes of fruit and vegetables, higher intakes of dietary fibre and lower intakes of fat [75]. Females are also however highly motivated by weight control and are more likely to diet or restrain their eating behaviour [75]. What is currently unknown however, is the driver behind the apparent differences in meal skipping between sexes and certain meals during young adulthood.

### Strengths and limitations

The present review has several strengths. It is the first attempt to bring together the literature on meal skipping correlates and employed a rigorous search strategy whilst adhering to the PRISMA protocol [27]. In addition, the use the Academy of Nutrition and Dietetics Quality Criteria Checklist by two independent reviewers to assess risk of bias [30], and the use of an established framework for reporting eating behaviour correlates [20], are regarded as strengths of this review.

An important limitation of this review is the lack of consistency in the terminology and definition of meal skipping and the measurement of these behaviours. Definitions of meal skipping varied; consuming three meals on <2 days/week, failing to report a meal in a food diary, to answering yes to “Do you skip meals?” This limitation is paralleled in the study of breakfast consumption [76], and meal patterns in general [77]. In addition, meal skipping was captured via differing methodologies, including food diaries, 24-h recalls, surveys and FFQ’s (which included a specially designed item to assess the daily consumption of meals and snacks). Each of these methods has its own strengths and weaknesses and are aimed at capturing dietary intake rather than the omission of eating occasions [78]. Questions designed to evaluate meal skipping were not consistent between studies, with continuous scales, binary and categorical responses utilised. These inconsistencies in definition and measurement limited our ability to compare the findings between studies. Furthermore, multiple methodological and reporting weaknesses were apparent in the reviewed articles. This was confirmed in our bias risk assessment, where 28 of the 35 studies scored a negative ranking. Results of the studies were poorly reported, with limited use of appropriate statistical analyses.

Another limitation of this review is the classification of young adults by age. Some included studies included participants outside of the 18–30 year age range, while the studies that reported university or college student samples failed to report the percentage of mature age students. Results therefore may not always be reflective of young adult populations.

Moreover, given that the majority of studies were cross-sectional, studies were not able to infer causation and thus the direction of the relationship for influences such as menstrual regulation and BMI was not clear. In addition, the generalisability of our findings may be limited by predominantly female populations and wide country-specific variations in religion, eating culture and socio-economic status. The subjectively ranked attributes of meal skipping provided a valuable insight into why young adults may skip meals. However, these results should be interpreted with caution due to the lack of clarity in the data collection methodology employed in these studies. For example, studies did not report if questions were open-ended or categorical, which may have impacted the results. It was also unclear in many cases if these questions were framed in terms of meal skipping in general or if it was directed at a specific meal (e.g. the breakfast meal), which is important given that meal skipping correlates appear to vary between meals.

### Implications for future research

This review highlighted several implications for future research. Firstly, definitions used to identify meal skipping are inconsistent. A standardised approach to defining meal skipping would provide clarity and allow for more reproducible results across studies. Secondly, the measurement of meal skipping in existing studies is inconsistent. With a high number of methods used to quantify meal skipping identified by this review, there is a need to standardise measurement so that more informative comparisons can be made. Thirdly, many of the reported correlates were within the intrapersonal domain of the SEF. This highlights the need to assess associations between correlates outside this domain such as physical environmental influences, to further examine why young adults are partaking in this unhealthy eating behaviour. Fourthly, this review focused only on the young adult population, future reviews should be conducted to understand correlates of meal skipping in different population groups e.g. elderly or child populations. Lastly, only four of the 35 studies had a positive risk of bias assessment score indicating that future nutrition research needs stronger design and reporting strategies. The Strobe-NUT [79] reporting guidelines are aimed at improving the reporting of observational studies with a focus on diet and health and should be employed in future research to increase transparency and consistency of nutritional epidemiology studies.

## Conclusions

This systematic review addressed a gap in the literature on the correlates of meal skipping in young adults. Results are consistent with previous research reporting that the breakfast meal is the most commonly skipped meal for this age group. This review highlights the perceived lack of time to be an important correlate of meal skipping. The sex of an individual was also reported to be an important correlate of meal skipping, with males more likely to skip breakfast and females more likely to skip lunch or dinner. Therefore, sex and meal specific components, and improvements in time management skills, may warrant further investigation as effective strategies for interventions targeting meal skipping in young adults.

## Additional files

Additional file 1: PRISMA 2009 Checklist. (DOC 68 kb)
Additional file 2: Table S2. Risk of bias assessment of included articles according to the Academy of Nutrition and Dietetics Quality Criteria Checklist [30]. (DOCX 57 kb)

## Abbreviations

BMI: Body mass index
FFQ: Food frequency questionnaire
PRISMA: Preferred Reporting Items for Systematic Reviews and Meta-Analysis
SCT: Social Cognitive Theory
SEF: Social-ecological framework
