# Supplementary material for: Correlates of meal skipping in young adults: a systematic review
Source: Int J Behav Nutr Phys Act. 2016 Dec 1;13:125. doi: 10.1186/s12966-016-0451-1 (PMC5133750; doi:10.1186/s12966-016-0451-1)
Supplement: Additional file 2: Table S2. — Risk of bias assessment of included articles according to the Academy of Nutrition and Dietetics Quality Criteria Checklist [30]. (DOCX 57 kb) [file 12966_2016_451_MOESM2_ESM.docx]

**Additional file 2: Table S2. Risk of bias assessment of included articles according to the Academy of Nutrition and Dietetics Quality Criteria Checklist [30]**

| **Article** | **1** | **2** | **3** | **4** | **5** | **6** | **7** | **8** | **9** | **10** | **Classification** |
| --- | --- | --- | --- | --- | --- | --- | --- | --- | --- | --- | --- |
| **Afolabi et al. 2013 [34]** | 1 | 1 | 0 | 1 | 0 | 1 | 0 | 0 | 0 | 0 | - |
| **Akarslan et al. 2008 [61]** | 1 | 1 | 1 | 0 | 0 | 1 | 0 | 1 | 1 | 0 | Ø |
| **Aryee et al. 2013 [35]** | 1 | 1 | 0 | 0 | 0 | 1 | 0 | 0 | 0 | 1 | - |
| **Bahl et al. 2013 [36]** | 1 | 0 | 0 | 0 | 0 | 0 | 0 | 1 | 1 | 0 | - |
| **Beerman et al. 1990 [62]** | 1 | 1 | 0 | 1 | 0 | 1 | 0 | 0 | 1 | 0 | Ø |
| **Chung et al. 2003 [37]** | 1 | 0 | 0 | 0 | 0 | 1 | 0 | 0 | 0 | 0 | - |
| **Colic Baric et al. 2003 [38]** | 1 | 1 | 0 | 0 | 0 | 1 | 0 | 0 | 0 | 1 | - |
| **Danquah et al. 2010 [39]** | 1 | 1 | 0 | 0 | 0 | 0 | 0 | 0 | 0 | 0 | - |
| **Deepika 2015 [40]** | 1 | 0 | 0 | 0 | 0 | 0 | 0 | 0 | 0 | 0 | - |
| **Eittah 2014 [41]** | 1 | 1 | 0 | 0 | 0 | 1 | 0 | 0 | 1 | 0 | - |
| **Eldisoky, 2003 [42]** | 1 | 1 | 0 | 0 | 0 | 0 | 0 | 0 | 0 | 0 | - |
| **Evagelou et al. 2014 [43]** | 1 | 0 | 0 | 0 | 0 | 1 | 0 | 0 | 1 | 0 | - |
| **Freedman 2010 [63]** | 1 | 1 | 0 | 1 | 0 | 1 | 1 | 0 | 1 | 1 | Ø |
| **Huang et al. 1994 [44]** | 1 | 1 | 0 | 0 | 0 | 0 | 1 | 0 | 1 | 0 | - |
| **Kapinos & Yakusheva 2011 [31]** | 1 | 1 | 1 | 1 | 0 | 1 | 1 | 1 | 1 | 1 | + |
| **Kim et al. 2010 [45]** | 1 | 0 | 1 | 1 | 0 | 1 | 1 | 1 | 1 | 0 | - |
| **Lamia Dhia & Ban Faud 2014 [46]** | 1 | 1 | 0 | 1 | 0 | 1 | 0 | 0 | 0 | 0 | - |
| **Laska et al. 2010 [1]** | 1 | 1 | 1 | 1 | 0 | 1 | 1 | 1 | 1 | 1 | + |
| **Lee & Yoon 2014 [47]** | 1 | 0 | 0 | 1 | 1 | 0 | 0 | 0 | 0 | 0 | - |
| **Musaiger & Radwan 1995 [48]** | 1 | 0 | 0 | 0 | 0 | 1 | 1 | 0 | 0 | 0 | - |
| **Neslisah & Emine 2011 [49]** | 1 | 1 | 0 | 0 | 0 | 1 | 0 | 0 | 0 | 0 | - |
| **Nicklas et al. 1998 [12]** | 1 | 0 | 1 | 1 | 0 | 1 | 1 | 1 | 1 | 0 | - |
| **Nzeagwu & Akagu 2011 [50]** | 1 | 0 | 0 | 0 | 0 | 1 | 1 | 0 | 0 | 0 | - |
| **Ozilgen 2011 [51]** | 1 | 0 | 0 | 0 | 0 | 1 | 0 | 0 | 0 | 0 | - |
| **Sakamaki et al. 2005 [52]** | 1 | 0 | 0 | 1 | 0 | 0 | 0 | 0 | 0 | 0 | - |
| **Sato-Mito et al. 2011 [32]** | 1 | 1 | 1 | 1 | 0 | 1 | 1 | 1 | 1 | 0 | + |
| **Shimbo et al. 2004 [53]** | 1 | 0 | 0 | 0 | 0 | 1 | 1 | 0 | 1 | 0 | - |
| **Suliburska etal. 2012 [54]** | 1 | 0 | 0 | 0 | 0 | 1 | 0 | 0 | 0 | 0 | - |
| **Suliga et al. 2012 [55]** | 1 | 0 | 0 | 0 | 0 | 1 | 0 | 0 | 0 | 0 | - |
| **Tanaka et al. 2008 [33]** | 1 | 1 | 1 | 0 | 0 | 1 | 1 | 1 | 1 | 0 | + |
| **Tominaga et al. 2012 [56]** | 1 | 0 | 0 | 0 | 0 | 1 | 1 | 0 | 1 | 0 | - |
| **Ukegbu et al. 2015 [57]** | 1 | 1 | 0 | 0 | 0 | 1 | 1 | 0 | 0 | 0 | - |
| **Yahia et al. 2008 [58]** | 1 | 0 | 0 | 0 | 0 | 1 | 1 | 0 | 1 | 0 | - |
| **Yildiza et al. 2011 [59]** | 1 | 0 | 0 | 0 | 0 | 0 | 0 | 0 | 0 | 0 | - |
| **Yilmaz et al. 2014 [60]** | 1 | 0 | 0 | 0 | 0 | 1 | 1 | 0 | 1 | 1 | - |

Criteria: 1) The study clearly stated the research question; 2) the selection of participants was free from bias; 3) if study groups were comparable; 4) participant withdrawal process documented; 5) the use of blinding was documented; 6) was participant compliance measured; 7) the measurements used were valid and reliable; 8) appropriate statistical analysis used; 9) biases and limitations documented; 10) funding or sponsorship reported [30].

1=Yes; 0=No or Unclear

MINUS/NEGATIVE (-) If most (six or more) of the answers to the above validity questions are “No,” the report should be designated with a minus (-) symbol on Evidence Worksheet.

NEUTRAL (Ø) If the answers to validity criteria questions 2, 3, 6, and 7 do not indicate that the study is exceptionally strong, the report should be designated with a neutral (Ø) symbol on the Evidence Worksheet.

PLUS/POSITIVE (+) If most of the answers to the above validity questions are “Yes” (including criteria 2, 3, 6, 7 and at least one additional “Yes”), the report should be designated with a plus symbol (+) on the Evidence Worksheet.
